# Activated glutamatergic neurons in basolateral amygdala suppress nicotine preference behavior in mice

**DOI:** 10.3389/fphar.2026.1807033

**Published:** 2026-04-30

**Authors:** Naotaka Izuo, Takuma Fujii, Takashi Asano, Yusuke Yano, Naoya Nishitani, Katsuyuki Kaneda, Atsumi Nitta

**Affiliations:** 1 Department of Pharmaceutical Therapy and Neuropharmacology, Faculty of Pharmaceutical Sciences, University of Toyama, Toyama, Japan; 2 Research Center for Advanced Science and Technology, The University of Tokyo, Tokyo, Japan; 3 Laboratory of Molecular Pharmacology, Institute of Medical, Pharmaceutical and Health Sciences, Kanazawa University, Kanazawa, Japan

**Keywords:** addiction, basolateral amygdala, chemogenetics, glutamatergic neurons, nicotine, smoking, two-bottle test

## Abstract

Despite the knowledge of the health risks of smoking, exposure to nicotine leads to smoking habits. Clarifying the neuronal mechanism of smoking preference formation underlying the development of smoking habits has a great impact on the establishment of effective smoking cessation therapies. In this study, the brain regions that determine the preference for oral nicotine intake in the two-bottle test were investigated. The activity of glutamatergic neurons reduced in the basolateral amygdala (BLA) of mice with a high preference for nicotine. Chemogenetic activation of glutamatergic neurons in the BLA decreased nicotine intake preference. These results indicate that glutamatergic neurons in the BLA modulate the preference for nicotine. This study presents a potential treatment strategy for smoking cessation that involves regulating the activity of glutamatergic neurons in the BLA.

## 1 Introduction

Tobacco smoking is a major global health risk factor, contributing to approximately 8 million deaths annually, which are caused by various cancers, cardiovascular diseases, and respiratory diseases. Furthermore, over 80% of smokers begin smoking before the age of 25 (Reitsma et al., 2021) and social peer pressure and other factors often serve as triggers for smoking (Barrington-Trimis et al., 2020). Despite the presence of negative perceptions, such as health risks during the early stages of smoking, repeated smoking experiences lead to a shift in preferences, with smokers increasingly perceiving positive effects, such as mood improvement and relaxation (Colvin and Mermelstein, 2010). This shift in preferences is underpinned by adaptive changes in brain circuits and functional alterations related to smoking behavior, which form the neural basis for the formation and maintenance of smoking habits as well as the difficulty of quitting. Nicotine, a component of tobacco, is rapidly incorporated into the brain after smoking and is deeply involved in the neuronal effects of tobacco. Clarifying the neural circuitry underlying preference changes in the formation of smoking habits, focusing on nicotine, is essential for elucidating the mechanisms of smoking dependence and developing effective smoking cessation support methods.

Human imaging studies on smoking habit formation have shown that the prefrontal cortex, insular cortex, cingulate gyrus, striatum, basolateral amygdala (BLA), hippocampus, and thalamus play important roles (Due et al., 2002; Engelmann et al., 2012; Janes et al., 2012; Lin et al., 2020). These regions are involved in diverse functions related to the acquisition and maintenance of smoking habits, including reward processing, decision making, emotional regulation, memory, and self-control. Studies using rodents have shown that the ventral tegmental area (VTA) and nucleus accumbens (NAc), which form the core of the reward system, are essential for the reinforcing effects of nicotine and the control of self-administration behavior (Fisher et al., 2021; Zhu et al., 2024), and that the medial prefrontal cortex (mPFC) is involved in nicotine-related reward learning (Zhu et al., 2024). In this study, we evaluated the preference of mice for nicotine-containing water using a two-bottle test. Initially, the mice did not prefer nicotine-containing water, however, after 1 week of forced exposure to nicotine-containing water, they began to show preference. Therefore, in this study, to clarify the neural basis of nicotine intake preference, we conducted a detailed analysis of preference changes using the two-bottle test and identified BLA as the brain region involved in preference behavior using chemogenetic methods.

## 2 Materials and methods

### 2.1 Animals

Male C57BL/6J mice (Nihon SLC, Shizuoka, Japan) aged 6–10 weeks were used for the experiments. The mice were housed in a regulated environment (25 °C ± 1 °C; 50% ± 5% humidity) with a 12 h-light/dark cycle (lights on at 7:00 a.m). Food and water were provided *ad libitum*. All experiments followed the National Institutes of Health Guidelines for the Care and Use of Laboratory Animals and were approved by the Committee for Animal Experiments of the University of Toyama (A2020PHA-11 and A2023PHA-14) and the Board of Safety Committee for Recombination DNA Experiments of the University of Toyama and Kanazawa University (G2020PHA-5 and G2020PHA-12).

### 2.2 Two bottle test

Two drinking water bottles were placed in each home cage. For habituation, mice at the age of 8–9 weeks were allowed to drink tap water from both bottles for 3 days, followed by 2% saccharin sodium dihydrate (Fujifilm, Osaka, Japan)-containing tap water from both bottles for following three successive days. For nicotine priming, the mice were exposed to 75 μg/mL nicotine-HCl (Merck, Darmstadt, Germany) containing saccharine solution from both bottles for 7 days following habituation as the nicotine priming phase. After nicotine priming, the mice were presented with a bottle containing 2% saccharine solution and another bottle containing 75 μg/mL nicotine in saccharine solution for 7 days as a preference test.

### 2.3 Immunohistochemistry

Immunohistochemistry was performed according to the standard protocol (Kusui et al., 2024). Immediately after the two-bottle test was performed six hours later from the dark phase, mice were anesthetized using a combination of anesthetics and perfused with ice-cold phosphate-buffered saline (PBS) followed by 4% paraformaldehyde (PFA) for fixation. The brains were collected, soaked in 4% PFA overnight, and subsequently in sucrose solutions (10%, 20%, and 30%). The brains were embedded by Tissue-Tek® O.C.T Compounds (Sakura Finetek Japan, Tokyo, Japan) and cut into 20 μm sections by Cryostat (CM3050S, Leica, Wetzlar, Germany). Sections were treated with 10 mM citrate solution (pH 6.0) for antigen retrieval at 100 °C for 20 min, washed with PBST, and then blocked in 5% goat serum (Merck) in PBST for 1 h. Sections were incubated with primary antibodies (anti-FosB (1:150, clone 5G4, Cell Signaling Technology, Danvers, MA, USA), anti-CaMKII (1:50, clone 6G9, Cell Signaling Technology), anti-PV (1:1000, clone PARV-19, Merck), and anti-SST (1:100, clone YC7, Abcepta, San Diego, CA, USA)) with 5% goat serum in PBST at 4 °C overnight. After washing with PBST, sections were incubated with secondary antibodies at room temperature for 2 h. After washing, the sections were incubated with 4′,6-diamidino-2-phenylindole (DAPI) (1:1000, Merck) in PBST for 15 min. After washing, sections were mounted using Fluoromount (Diagnostic BioSystems, Pleasanton, CA, USA). Images were captured using a microscope (BZ-X800; KEYENCE, Osaka, Japan). The number of FosB-positive cells and the area of each region were analyzed using BZ-X analyzer software (KEYENCE).

### 2.4 Chemogenetics

Chemogenetics enables the selective control of neuronal activity through the combined use of designed receptors and their cognate synthetic ligands. Expression of the receptor alone, as well as oral administration of a highly selective ligand such as deschloroclozapine (DCZ), has been experimentally demonstrated to exert minimal off-target or nonspecific effects on neuronal activity (Nagai et al., 2020; Suthard et al., 2023). Under anesthesia by a combination of anesthetics, AAV-CaMKII-hM3Dq-mCherry (Esaki et al., 2025) were administered bilaterally into the central region of BLA (anteroposterior, −1.4 mm; mediolateral, ±3.3 mm; dorsoventral, 4.9 mm) (The Mouse Brain in Stereotaxic Coordinates, 2026) of mice at 6 weeks of age. All animals receiving microinjection were applied to the experiments as the spread of the injected solution was confined to the BLA and did not extend to adjacent brain regions (Supplementary Figure S1). Fifteen days after microinjection, a two-bottle test was performed. DCZ (Hello Bio, Bristol, UK), a selective ligand for hM3Dq, was added to the drinking solution in both bottles at a concentration of 0.189 μg/mL to achieve a body weight of approximately 30 μg/kg (Suthard et al., 2023).

### 2.5 Statistical analyses

Experimental data were analyzed using Prism version 10 (GraphPad Software, La Jolla, CA, USA). The results are presented as mean ± standard error of the mean (SEM). For statistical analysis, Student’s t-test was used to compare two groups after confirming normality and homogeneity of variances and the chi-squared test was used for comparisons of proportions; in all cases, *p* < 0.05 was considered significant.

In Figure 2b, one data plot with a large deviation from the high-preference group in the BLA and the low-preference group in the PFC was omitted by performing Smirnov–Grubbs analysis.

## 3 Results

### 3.1 Nicotine priming increases the preference for the nicotine-containing bottle

**FIGURE 1:**
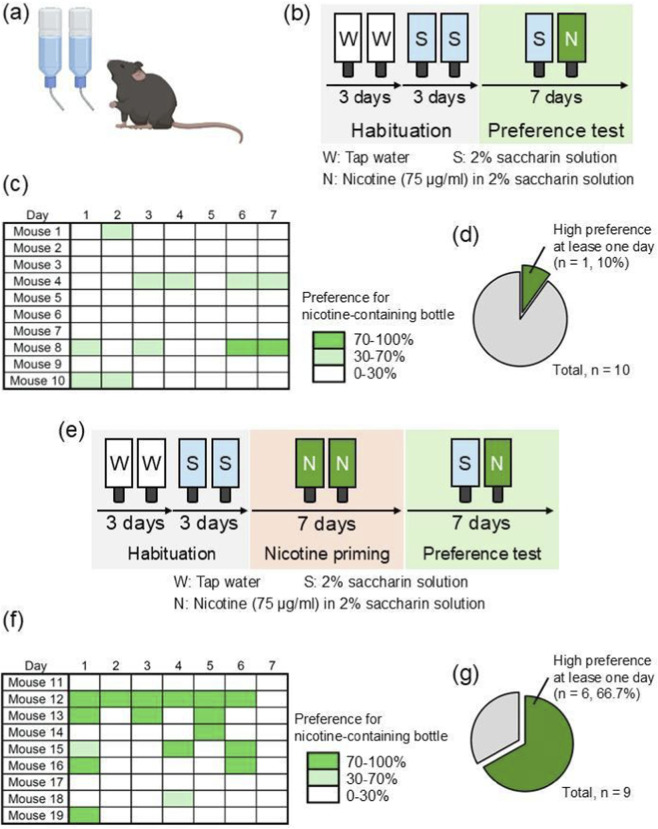
Nicotine priming increases the preference for the nicotine-containing bottle. **(a,b,e)** Illustration of the experimental design and schedule of the two-bottle test. In the two-bottle test without nicotine priming **(b)**, one in ten mice (10%) exhibited high preference to the nicotine-containing bottle at least 1 day **(c,d)**. In the two-bottle test with seven-day nicotine priming **(e)**, six in nine mice (66.7%) exhibited high preference to the nicotine-containing bottle at least 1 day **(f,g)**. Higher ratio of the mice with high preference to the nicotine-containing bottle were observed under the condition with nicotine priming than that without (*p = 0.0106) **(d,g)**. Statistical analysis was performed by chi-squared test for comparisons of proportions.

The preference for nicotine-containing bottles with or without nicotine priming was compared using the two-bottle test in the home cage. Immediately after habituation, mice were allowed to drink tap water from both bottles for 3 days and then 2% saccharine-containing tap water from both bottles for the following 3 days. Further, one bottle of 2% saccharine water and the other bottle containing 75 μg/mL nicotine in the saccharine water were presented to the mice for 7 days as a preference test. In this experimental procedure without nicotine priming, one in ten mice (10%) exhibited a high preference (>70%) for the nicotine-containing bottle for at least 1 day during the preference test (Figures 1a–d). In contrast, in the experimental course with nicotine priming, in which mice were forced to drink 75 μg/mL nicotine-containing water with 2% saccharine from both bottles for 7 days following habituation, six of the nine mice (66.7%) showed a high preference for the nicotine-containing bottle during the preference test (Figures 1e–g). A higher ratio of mice with a high preference for the nicotine-containing bottle was observed under the nicotine priming condition (chi-square test, *p = 0.0106). Through the two-bottle test, water intake showed similar pattern between the group with high and low preference to the nicotine-containing bottle (Supplementary Figure S2). This suggests that nicotine priming increases the preference for nicotine-containing bottles.

### 3.2 Identification of the neurons related to the preference to the nicotine bottle

**FIGURE 2:**
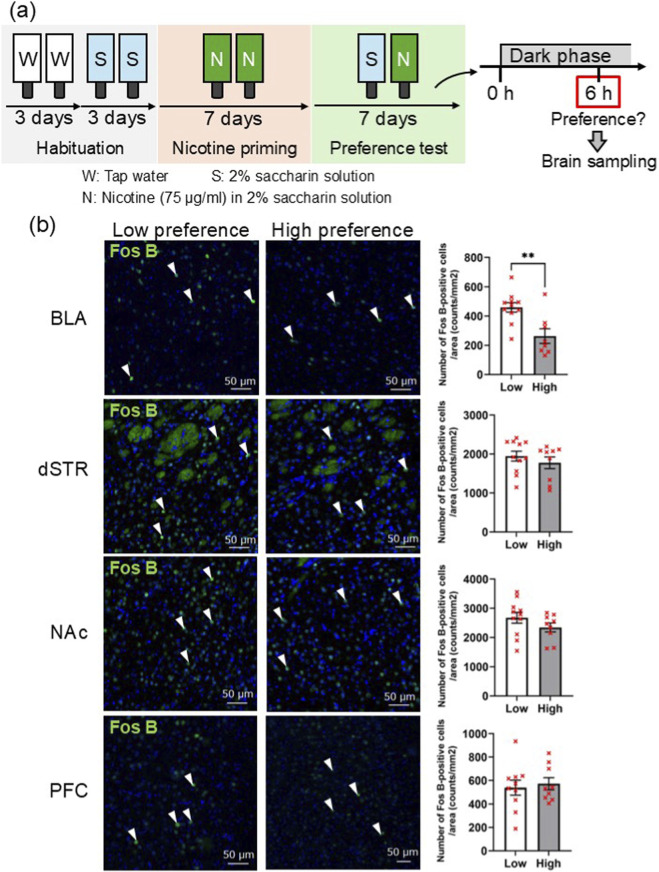
Lower number of FosB-positive cells was observed in basolateral amygdala (BLA) in the mice with high preference to the nicotine-containing bottle. **(a)** Illustration of the experimental and schedule of the two-bottle test with seven-day nicotine priming followed by brain sampling for immunohistochemical staining. Mice with high preference (>70%) and the same or more numbers of those with lower preference (<30%) were sacrificed six hours after the start of dark phase. **(b)** Immunohistochemistry to FosB, long-lasting neuronal activity marker, in the mice with high preference (n = 9) and those with low (n = 11) revealed that lower numbers of activated neurons were detected in the BLA (**p = 0.0029), not in the nucleus accumbens (NAc) (p = 0.1953), dorsal striatum (dSTR) (p = 0.3935), or prefrontal cortex (PFC) (p = 0.6919). Results are presented as mean ± standard error of the mean (SEM). Statistical analysis was performed by Student’s t-test after confirming normality and homogeneity of variances. In Figure 2b, one data plot with a large deviation from the high preference group in the BLA and low preference group in the PFC was omitted by performing a Smirnov–Grubbs analysis.

**FIGURE 3:**
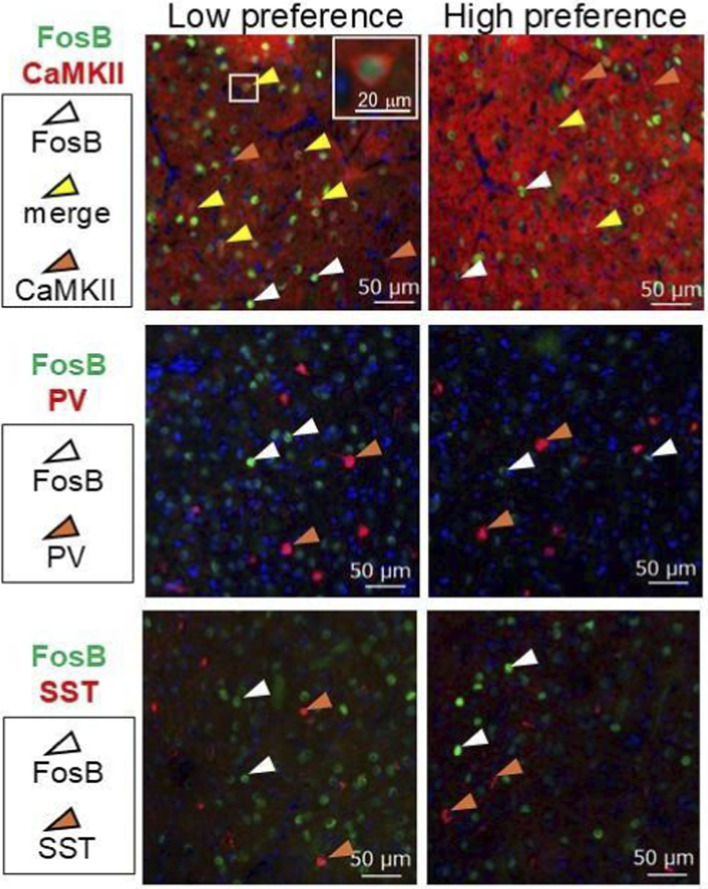
FosB-positive cells in the BLA were identified as CAMKII-positive glutamatergic neurons. Immunohistochemical staining was performed to identify FosB-positive neurons in the BLA. Immunosignals of FosB merged exclusively with those of calcium-calmodulin-dependent protein kinase II (CaMKII), a marker of glutamatergic neurons, though not with those of parvalbumin (PV) or somatostatin (SST), markers of GABAergic neurons. Nuclear counterstaining was performed with DAPI.

To clarify the neurological mechanism of the preference for the nicotine-containing bottle, the brains of mice that exhibited a high preference six hours after the start of the dark phase were collected. In addition to mice with a high preference (>70%), the same or more mice with a low preference (<30%) were randomly selected for sacrifice. By immunohistochemical analysis of FosB, a long-lasting neuronal activity marker, regional activation in the NAc, dorsal striatum (dSTR), prefrontal cortex (PFC), and BLA, which are involved in addiction, were compared (Figure 2a). No difference of the density of FosB-positive cells between mice with high and low preference were observed in the NAc (low preference, 2677.74 ± 185.34 counts/mm^2^, n = 11; high preference, 2345.29 ± 152.06 counts/mm^2^, n = 9, *p* = 0.1953), dSTR (low preference, 1946.80 ± 126.86 counts/mm^2^, n = 11; high preference, 1775.85 ± 150.74 counts/mm^2^, n = 9, *p* = 0.3935), or PFC (low preference, 539.53 ± 63.28 counts/mm^2^, n = 10; high preference, 572.79 ± 51.09 counts/mm^2^, n = 9, *p* = 0.6919), whereas significantly a greater number of FosB-positive cells was observed in the BLA of the mice with low preference (low preference, 459.41 ± 32.16 counts/mm^2^, n = 11; high preference, 262.86 ± 49.83 counts/mm^2^, n = 8, **p = 0.0029) (Figure 2b). Next, the types of neurons that were activated in the BLA of mice without preference were explored. BLA contains parvalbumin (PV)- and somatostatin (SST)-positive interneurons and calcium calmodulin-dependent protein kinase II (CaMKII)-positive glutamatergic neurons (Wolff et al., 2014). FosB-positive cells exclusively merged with glutamatergic neurons, though not with GABAergic interneurons (Figure 3). These results suggest that glutamatergic neurons in the BLA are involved in the regulation of nicotine bottle preference.

### 3.3 Chemogenetic activation of glutamatergic neurons in the BLA suppressed the preference to the nicotine-containing bottle figures

**FIGURE 4:**
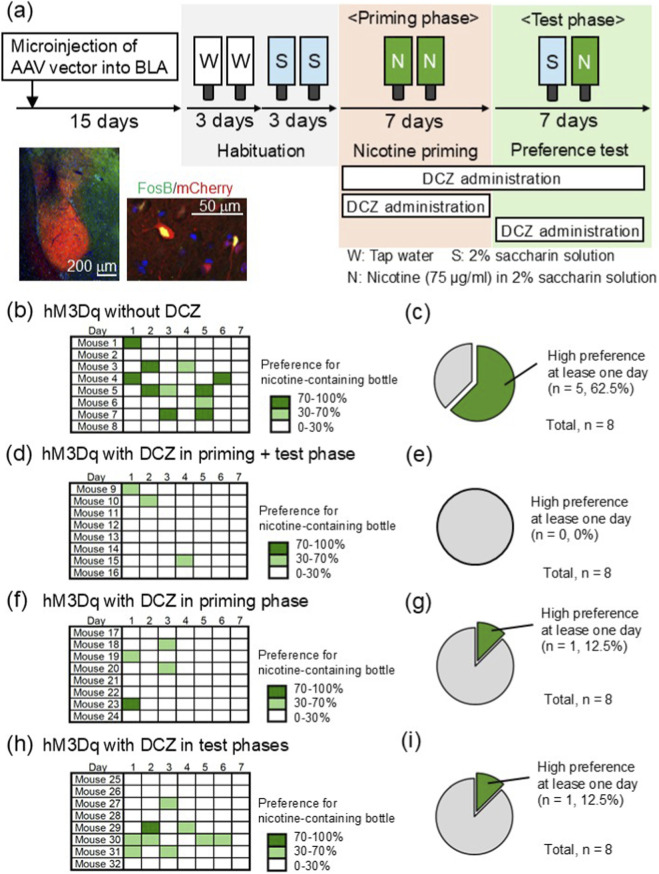
Chemogenetic activation of glutamatergic neurons in the BLA suppressed the preference to the nicotine-containing bottle. **(a)** Illustration of the experimental and schedule of the two-bottle test with virus microinjection and seven-day nicotine priming. Infection of adeno-associated virus (AAV) was confirmed by mCherry fluorescence 3 weeks after AAV microinjection. Chemogenetic activation of glutamatergic neurons was performed by the overexpression of hM3Dq under CAMKII promoter by the AAV and oral administration of deschloroclozapine (DCZ), a selective agonist for hM3Dq, through drinking water. Comparing with the mice without DCZ administration (five in eight mice (62.5%) with high preference) **(b,c)**, those with DCZ both in the nicotine priming and preference test phases **(d,e)**, just in the nicotine priming phase **(f,g)**, or just in the preference test phase **(h,i)** exhibited lower preference to the nicotine-containing bottle ((none in eight mice, (0%), **p = 0.00700; one in eight mice, (12.5%), *p = 0.0389; one in eight mice, (12.5%), *p = 0.0389, respectively). (Chi-squared test, **p = 0.00700, *p = 0.0389, *p = 0.0389, respectively). Statistical analysis was performed by chi-squared test for comparisons of proportions.

To examine the regulation of nicotine bottle preference by glutamatergic neurons in the BLA, neuronal activity was modulated chemogenetically by hM3Dq, a designed receptor for activation, and DCZ, a selective agonist of hM3Dq. Three weeks after AAV injection into the BLA, FosB-positive population exhibited mCherry fluorescence under the CaMKII promoter (Figure 4a), which is consistent with Figure 3. Lowered ratio of mice with high preference (>70%) for the nicotine bottle at least 1 day during the preference test was observed in the group with the activation of glutamatergic neurons in the BLA by DCZ oral administration through drinking solutions during both nicotine priming and preference test phases (none in eight mice, 0%), just nicotine priming phase (one in eight mice, 12.5%), or just preference test phase (one in eight mice, 12.5%), compared with the group without activation (five in eight mice, 62.5%) (Chi-squared test, **p = 0.00700, *p = 0.0389, *p = 0.0389, respectively) (Figures 4b–i). Through the two-bottle test, water intake showed similar pattern among four different paradigm of DCZ administration (Supplementary Figure S3). These results suggest that activation of glutamatergic neurons in the BLA suppresses the preference for nicotine bottles.

## 4 Discussion

In this study, in a two-bottle test, mice initially showed less preference for the nicotine-containing bottle, probably related to the aversive response (Akinola et al., 2022); however, after 1 week of forced nicotine priming via drinking water, some mice showed a preference for nicotine-containing bottles. Fewer activated glutamatergic neurons were observed in the BLA in individuals with a high preference for nicotine-containing bottles than in those with a low preference. Chemogenetic activation of glutamatergic neurons in the BLA during the nicotine priming or preference test phases decreased the number of individuals exhibiting a preference for nicotine-containing bottles. These results indicate that the formation of a preference for the nicotine-containing bottle and its behavioral expression are modulated by glutamatergic neurons in the BLA.

The BLA plays an important role in controlling rewards, emotions, and addictive behaviors (Wassum and Izquierdo, 2015; Tokutake et al., 2022). Beyeler et al. (2018) revealed that positive- and negative-valence neurons are spatially intermingled within the BLA but are functionally biased according to their projection targets. Projections to the NAc and central amygdala (CeA) have been associated with reward and aversion-related behaviours, respectively. In parallel, Zhang et al. (2021) demonstrated that the BLA contains distinct neuronal populations that are intrinsically selective for positive or negative valence, which acquire corresponding cue responses through learning. These populations are embedded in segregated input–output circuits, with projections to the olfactory tubercle and nucleus accumbens preferentially mediating appetitive and aversive behaviours, respectively, suggesting that valence is organized at the level of defined cell types and circuits. Consistent with this view, valence representations in the BLA have also been shown to be encoded at the population level and dynamically remapped during learning, indicating that behavioral output reflects the collective activity of distributed neuronal ensembles rather than individual neuronal activity (Zhang and Li, 2018).

Together, these findings suggest that valence in the BLA is not determined along a single axis but arises from multiple levels of organization. Although positive and negative representations coexist within the same region, they are structured by both cell-type identity and projection-defined circuits, with valence direction determined by the combined influence of intrinsically valence-selective neuronal populations, their downstream projection targets, and state-dependent recruitment of these circuits.

In this framework, the transition from aversion to preference observed with repeated nicotine exposure can be interpreted as a shift in circuit engagement rather than a reversal of intrinsic value. While nicotine initially elicits aversive responses, repeated exposure may progressively recruit appetitive or reward-related circuits within the BLA. Thus, nicotine exposure is more likely to reflect the addition of appetitive circuit activity while aversive representations remain intact, consistent with a model in which parallel valence circuits coexist and behavioral output depends on their relative contribution.

The persistence of variability in nicotine preference further suggests that this process does not immediately stabilize into a single valence state. Instead, the system may remain in a transitional regime in which both aversive and appetitive circuits are active, and behavior reflects their dynamic balance. In this context, fluctuations in internal state like neuromodulatory tone may bias the relative recruitment of these circuits across sessions, resulting in the observed variability in preference.

In addition to circuit composition and recruitment, the functional state of amygdalo-striatal circuits may critically influence valence-related behavior. In particular, alterations in glutamatergic transmission within these circuits have been shown to modulate activity across the BLA, CeA, and striatum (Domi et al., 2023). Such changes in baseline excitatory tone could bias the relative engagement of valence-related pathways, thereby influencing behavioral output. In this context, the selective modulation of neuronal activity observed in the BLA, but not in other regions, may suggest that valence-related computations are preferentially regulated within the amygdala rather than uniformly across the circuit. Within this framework, variability in nicotine preference may reflect not only differences in circuit recruitment but also dynamic shifts in the excitatory state of these networks.

Although activation of BLA glutamatergic neurons suppressed nicotine preference in the present study, this effect may appear inconsistent with previous reports showing that reactivation of BLA neuronal ensembles promotes nicotine preference and relapse (Xue et al., 2017). However, these findings are not necessarily contradictory, as distinct neuronal ensembles within the BLA are known to encode different aspects of nicotine-associated memories, and behavioral outcomes may depend on which neuronal subsets are engaged. In addition, nicotinic acetylcholine receptors are expressed in BLA circuits and can modulate synaptic transmission and excitatory–inhibitory balance (Feduccia et al., 2012; Pidoplichko et al., 2013), suggesting that nicotine may influence circuit dynamics by altering glutamatergic signaling. Further studies incorporating more selective targeting strategies, together with direct assessment of nicotinic receptor–dependent synaptic mechanisms, will be required to clarify the specific neuronal populations and circuit processes underlying these effects.

This study clarified the role of glutamatergic neurons in the BLA associated with nicotine intake behavior and revealed that their activation suppresses nicotine preference. This study not only disclose the mechanism of smoking habit formation but also provides evidence for establishing smoking cessation therapy.

## Data Availability

The original contributions presented in the study are included in the article/Supplementary Material, further inquiries can be directed to the corresponding author.
