# Supplementary material for: Activated glutamatergic neurons in basolateral amygdala suppress nicotine preference behavior in mice
Source: Front Pharmacol. 2026 Apr 30;17:1807033. doi: 10.3389/fphar.2026.1807033 (PMC13171807; doi:10.3389/fphar.2026.1807033)
Supplement: Supplementary file 1 [file Supplementaryfile1.docx]

Supplementary Material

Supplementary Figures


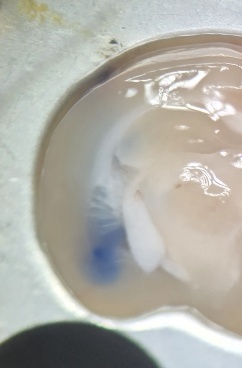
Fig.S1

**Supplementary fig. 1. Water intake during two-bottle test (related to fig. 1).**

Under anesthesia by a combination of anesthetics, dye was administered into the central region of BLA (anteroposterior, -1.4 mm; mediolateral, 3.3 mm; dorsoventral, 4.9 mm) of mice at 6 weeks of age.

Fig.S2


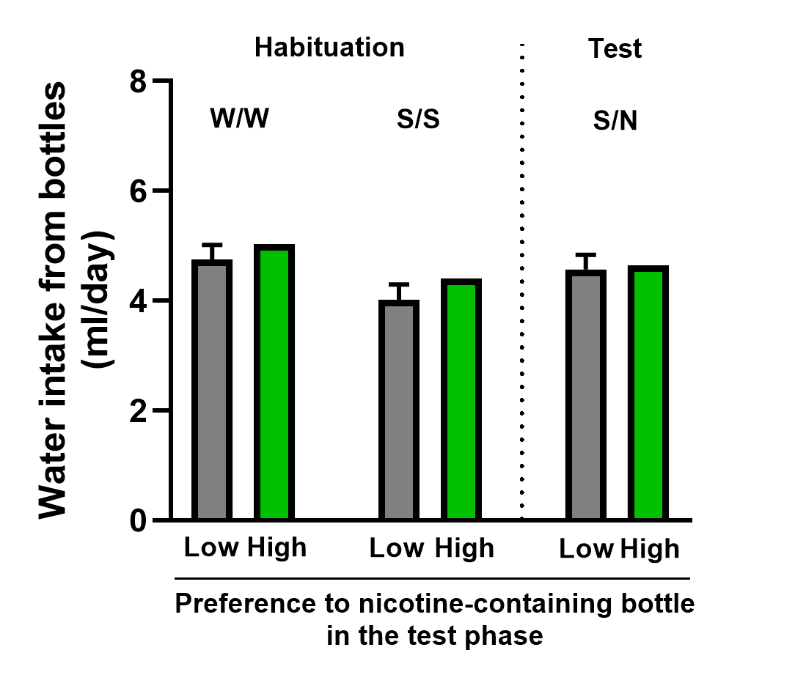

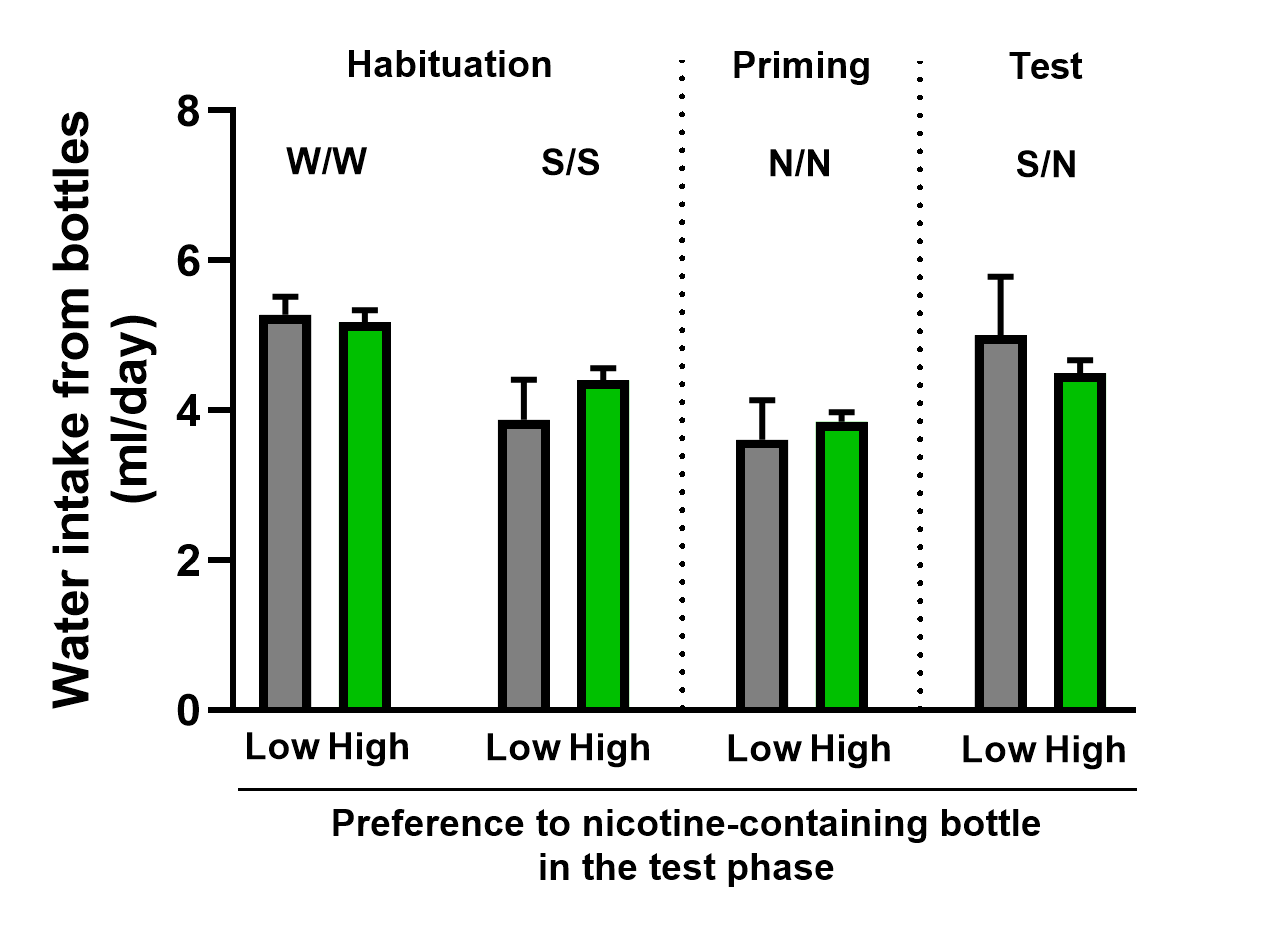


(a)

(b)

**Supplementary fig. 2. Water intake during two-bottle test (related to fig. 1).**

Water intake of each mouse in the group with high or low preference to the nicotine-containing bottle were shown as the average of each mouse per day with SEM

Fig.S3


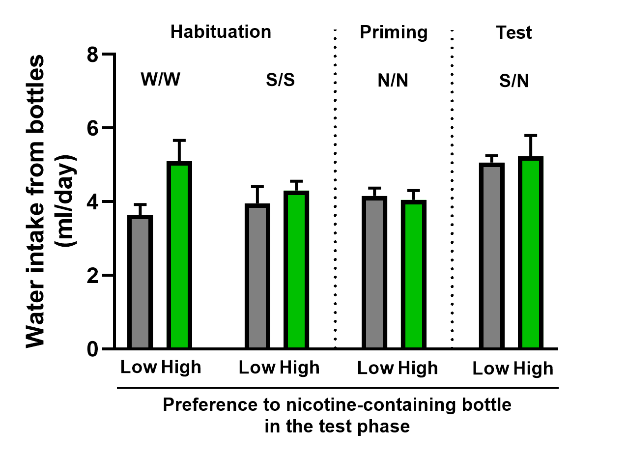

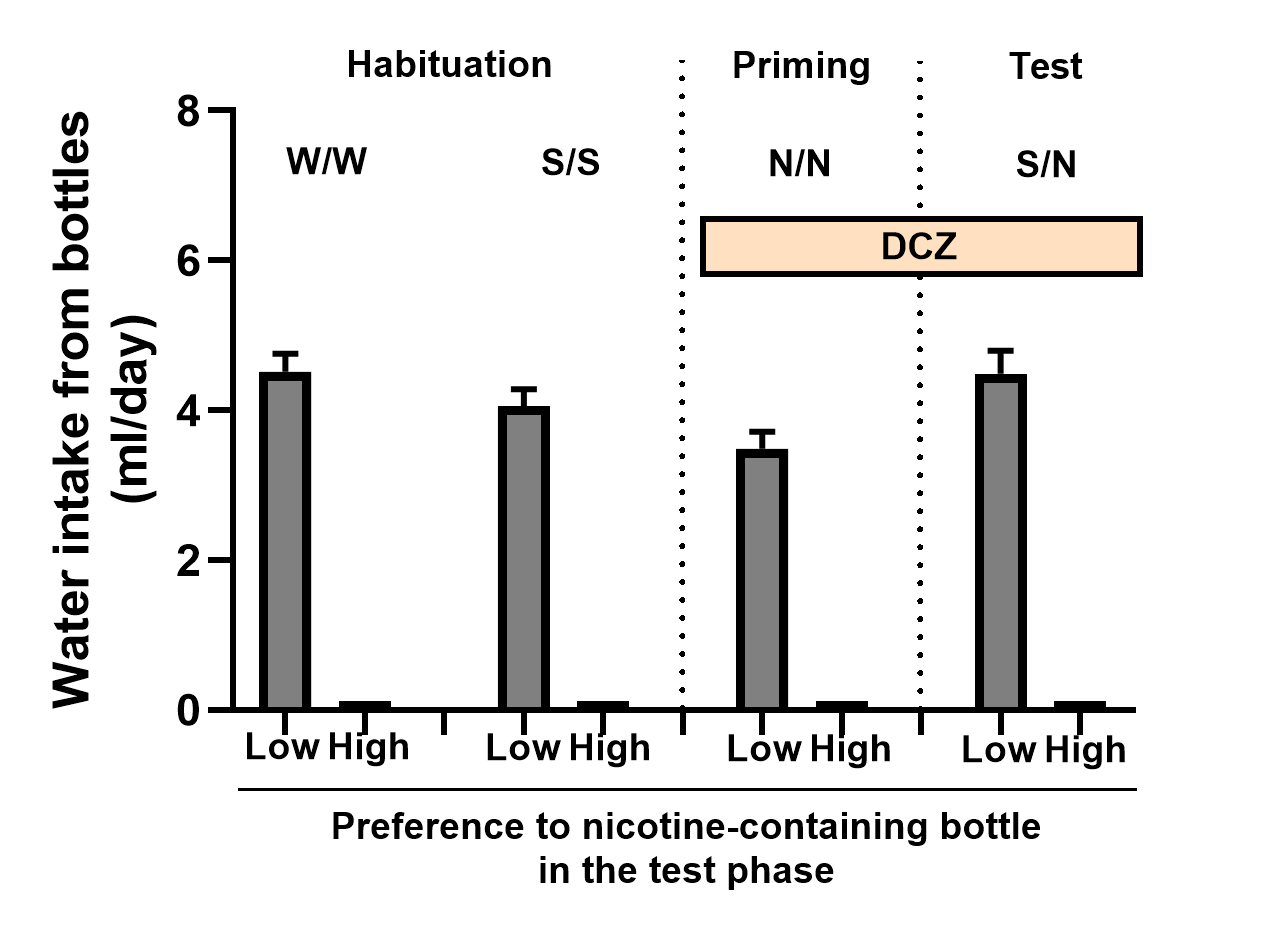

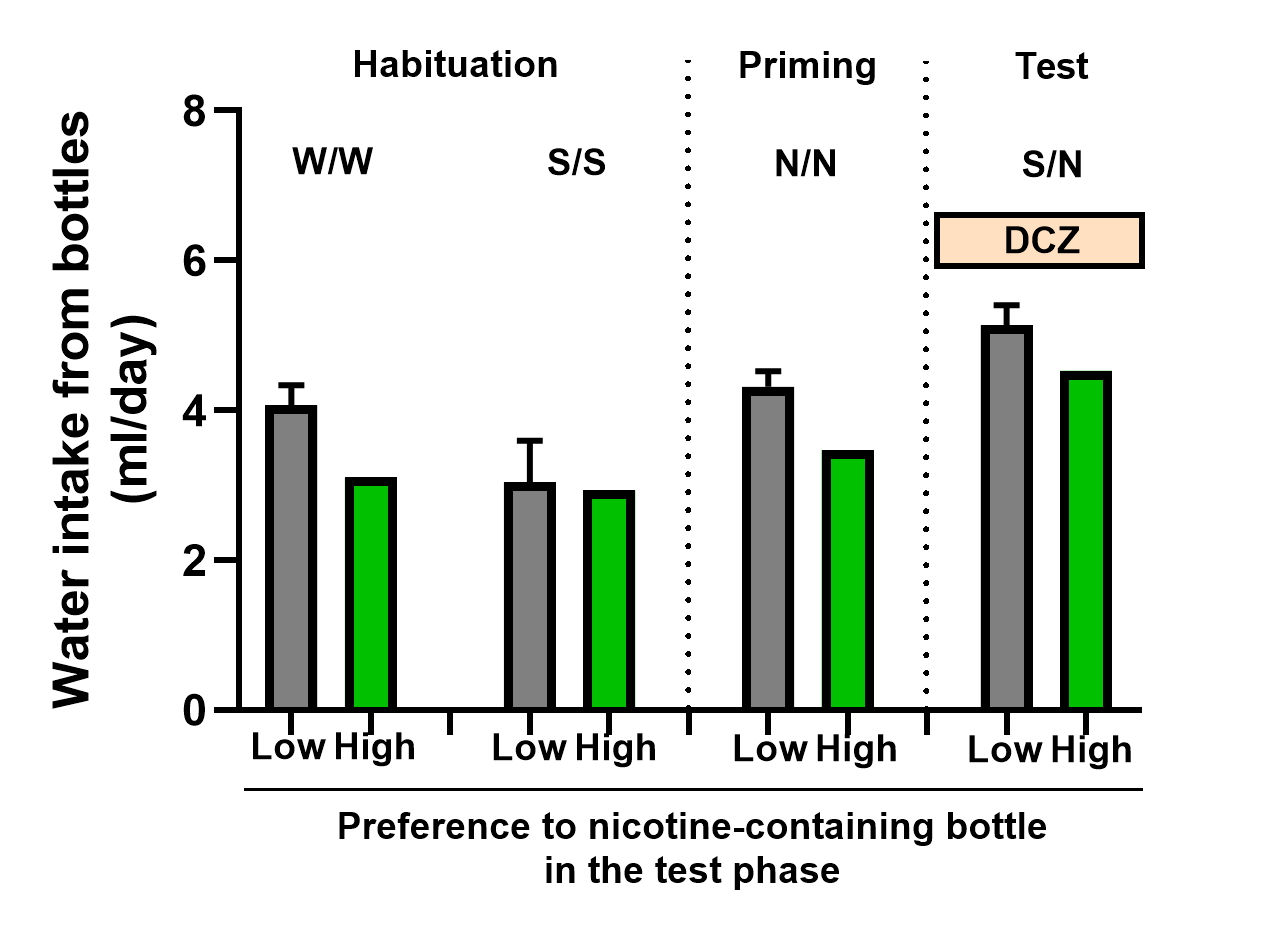

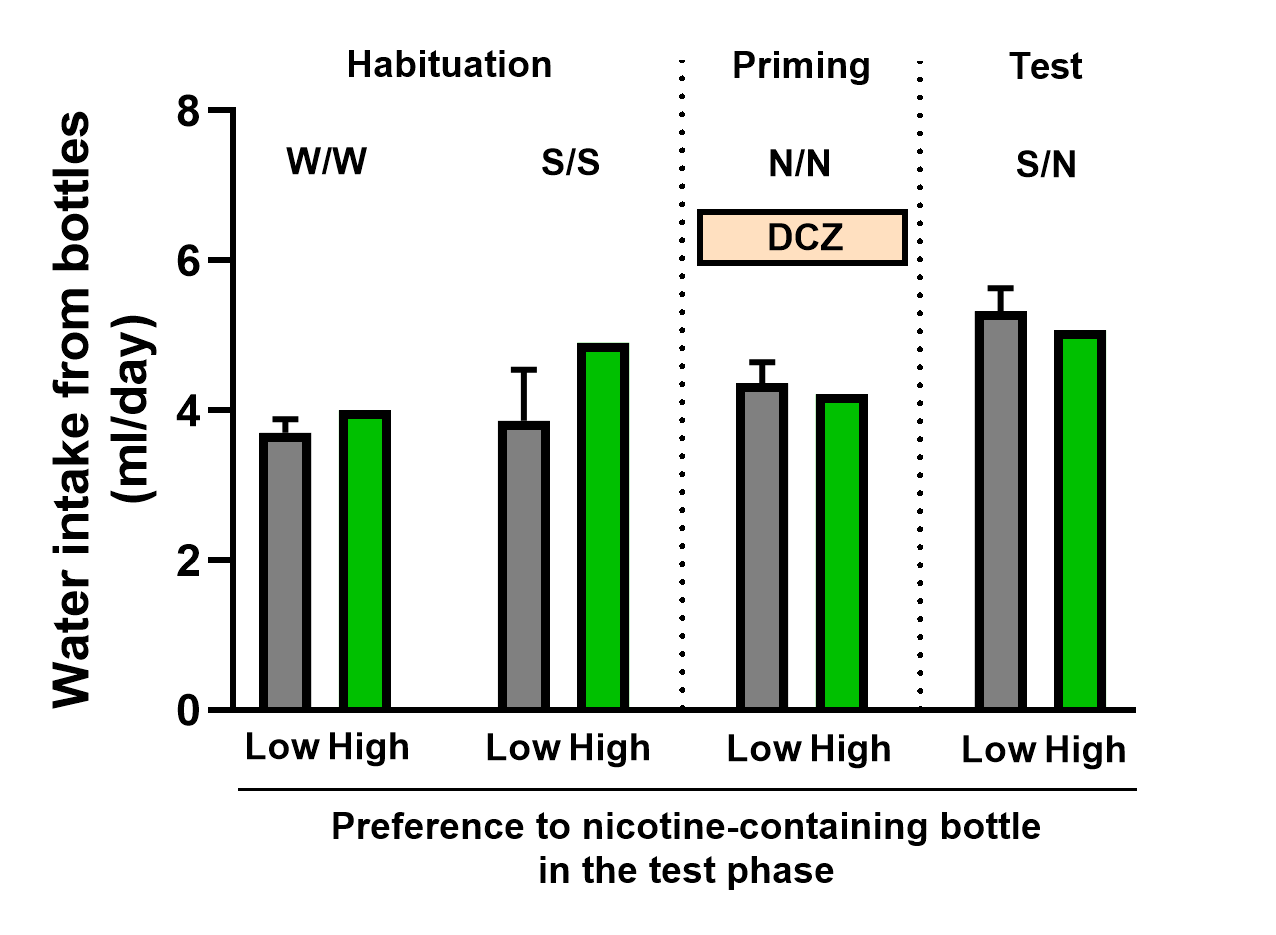


(a)

(b)

(c)

(d)

**Supplementary fig. 3. Water intake during two-bottle test with or without DCZ (related to fig. 4).**

Water intake of each mouse after AAV injection in the group with high or low preference to the nicotine-containing bottle were shown as the average of each mouse per day with SEM
